# Accounting for Withdrawal of Life-Sustaining Treatment in the Analysis of Traumatic Brain Injury Studies

**DOI:** 10.1089/neur.2025.0010

**Published:** 2025-05-27

**Authors:** Brian C. Healy, Brian L. Edlow, Yelena G. Bodien

**Affiliations:** ^1^ Ann Romney Center for Neurologic Diseases, Brigham and Women’s Hospital, Boston, Massachusetts, USA.; ^2^ Department of Neurology, Harvard Medical School, Boston, Massachusetts, USA.; ^3^ Massachusetts General Hospital Biostatistics Center, Massachusetts General Hospital, Boston, Massachusetts, USA.; ^4^ Center for Neurotechnology and Neurorecovery, Massachusetts General Hospital, Boston, Massachusetts, USA.; ^5^ Athinoula A. Martinos Center for Biomedical Imaging, Massachusetts General Hospital and Harvard Medical School, Boston, Massachusetts, USA.; ^6^ Department of Physical Medicine and Rehabilitation, Spaulding Rehabilitation Hospital and Harvard Medical School, Charlestown, Massachusetts, USA.

**Keywords:** biostatistics, traumatic brain injury, withdrawal of life-sustaining therapy

## Abstract

Studies that aim to evaluate outcomes after severe traumatic brain injury (TBI) must account for patients who die after withdrawal of life-sustaining treatment (WLST). If we are willing to assume that some of the patients who die of WLST might have had a good outcome at 6 months, the choice of analytic approach may impact the results. In this study, 6-month clinical outcomes for patients with TBI were simulated under six different scenarios related to WLST. Each scenario represents different assumptions related to the decision to choose WLST and how that decision relates to the 6-month clinical outcome. For each simulated dataset and scenario, three analytic approaches were used to estimate the probability of a good outcome at 6 months: complete case analysis, worst-case imputation, and inverse probability weighted analysis. The bias of the estimate from each of the approaches was used to compare the performance of the analysis approaches. When the probability of WLST was equal for all patients (i.e., covariates were not factored into the WLST decision), both the complete case analysis and the inverse probability weighted analysis were unbiased. When only patients who would have a poor outcome at 6 months were eligible to have WLST, only the worst-case imputation analysis was unbiased. When the probability of WLST was a function of observed patient characteristics that were also related to 6-month outcome (e.g., age, injury severity), only the inverse probability weighted analysis was unbiased. Finally, when the probability of missingness was related to an unobserved patient characteristic, none of the approaches were unbiased. If some patients who die of WLST might have had a good outcome, inverse probability weighting could be considered to decrease bias associated with censoring or imputing poor outcomes for participants with WLST.

## Introduction

After a patient experiences a severe traumatic brain injury (TBI), there are many potential recovery trajectories, and improvement in function may continue for at least a decade.^1,2^ One of the main challenges for families is that decisions regarding critical clinical care are made in the days following injury without precise knowledge about the degree to which survival and recovery are possible. Some families decide to withdraw life-sustaining treatment (WLST) in the intensive care unit, often as a result of a poor neurological prognosis provided by the clinical team.^3^ Although many patients who die due to WLST may have ultimately had a poor outcome, some of these patients might have recovered had life sustaining therapy (LST) been prolonged.^4^

In addition to the challenges facing patients and families, studies evaluating the natural history of recovery from TBI or developing prognostic models for the status of patients 6 months after a TBI must account for the “missing” 6-month outcome data from patients who die after WLST. The clinical status of a patient had LST been continued must be considered “missing data” from a statistical perspective because the outcome cannot be observed. In the presence of this “missing data,” there are several analysis approaches that can be followed, and each analysis approach is valid under specific assumptions regarding the missing data mechanism.

One analysis approach is to remove patients who died after WLST from the analysis and perform analyses only on the patients with complete, known outcomes. This is called a complete case analysis because only the patients with an observed 6-month outcome contribute to the analysis. Unfortunately, the complete case analysis would only be valid if the patients who die after WLST are a random sample of the original sample. This is called missing completely at random (MCAR) using the definitions of Little and Rubin.^5^ However, multiple studies suggest that WLST is associated with specific demographic and clinical characteristics, indicating these patients have a worse prognosis than patients who do not choose WLST.^6^ Thus, the assumption that patients who die after WLST represent a random sample of the original group is very likely incorrect.

A second approach is to assign all patients who die after WLST a poor outcome. Some researchers consider this the most appropriate approach since patients who die after WLST did have a poor outcome (i.e., death), and this poor outcome should be reflected in any statistical analysis. This could be considered a form of imputation from a statistical perspective because we are assigning subjects who died after WLST with the poor outcome. Although this approach is easy to implement in the analysis, several previous studies have indicated that assuming a poor outcome for all patients may overestimate the proportion who would have a poor outcome because some patients with severe injuries recover.^2,7^

If we are willing to assume that some of the patients who die of WLST might have had a good outcome at 6 months, modern statistical approaches, including inverse probability of censoring weights (IPW), have been developed to handle this type of missing outcome data.^8^ IPW uses all patients in a cohort to estimate the probability a patient will have WLST and then weights the outcome of patients who do not have WLST to account for the outcomes that were not observed for the patients who have WLST. IPW has been shown to be valid when the data are missing at random (MAR) as defined by Little and Rubin, which means that the probability of missing outcome data is related to observed patient characteristics.^5^ This assumption is a less strong assumption compared to MCAR, which means it may be true in more cases.^9^

In this study, we use simulated data under six different clinical scenarios to examine analysis approaches for handling WLST in TBI. The goal of the study is to understand how different assumptions regarding WLST impact statistical analysis. In particular, we will determine the scenarios where each analysis approach provides valid estimates. Further, we show that appropriate statistical analysis approaches can provide unbiased estimates of the probability of a good outcome at 6 months when the reason for choosing WLST is known.

## Methods

### Simulation study

We generated simulated datasets with parameters based on published data from the Transforming Research and Clinical Knowledge in TBI (TRACK-TBI) study, which enrolled patients with TBI across 18 United States level 1 trauma centers and conducted longitudinal follow-up for 12 months post-injury.^4,10^ The published data used for the simulation included only TRACK-TBI patients that had been admitted to the ICU. We simulated the six strongest previously published clinical predictors of WLST: age, day 0 best Glasgow Coma Scale (GCS) motor subscale score,^11^ presence of decompressive craniectomy, complications composite (sum of 54 complications), injury severity score total,^12^ and CT Rotterdam score.^4,13^ The characteristics of patients with WLST and without WLST from this prior study are provided in Table 1. The primary outcome measure was the Glasgow Outcome Scale-Extended (GOSE),^14^ an 8-category measure that ranges from death (score = 1) to recovery to pre-injury level of function (score = 8) that is the most frequently used outcome in TBI research.^15^ The GOSE score is frequently dichotomized into “poor outcome” (e.g., GOSE ≤3 indicating an outcome of death, vegetative state, or severe disability requiring nearly 24-h care) and “good outcome” (i.e., GOSE ≥4 indicating recovery to at least partial independence in the home).

**Table 1.** Characteristics of Patients with and without WLST in TRACK-TBI

	Patients without WLST	Patients with WLST
*N*	1302	90
Age (years; Mean [SD])	42.5 (18.1)	59.2 (17.9)
Day 0 best Glasgow Coma Scale motor subscale score (Mean [SD])	5.63 (0.89)	3.81 (1.59)
Presence of decompressive craniectomy (%)	14%	44%
Complications composite (Mean [SD])	1.45 (2.41)	3.38 (3.15)
Injury Severity Score total (Mean [SD])	19.4 (11.0)	26.9 (11.3)
CT Rotterdam score (Mean [SD])	2.79 (1.02)	4.22 (1.39)

TRACK-TBI, transforming research and clinical knowledge in traumatic brain injury; SD, standard deviation; WLST, withdrawal of life-sustaining treatment.

In the simulation, we generated 10,000 independent datasets with 500 patients in each dataset. For the 500 patients, the dichotomous outcome of 6-month GOSE score of ≥4, which we will define as a good outcome, was simulated for each of the patients based on seven predictors: the six predictors of WLST provided above and one additional predictor that was considered “unmeasured” to the study. The “unmeasured” predictor represents any other characteristic of the patient that is not, or cannot be measured in our data. All the predictors were standardized so that a one-unit increase in the predictor corresponded to a one-standard-deviation increase in the predictor, and all predictors were independent. The probability of a good outcome was simulated using a logistic model with coefficients of 0.5 (positive or negative depending on the covariate) for each of the seven predictors. Each of the predictors was included in the logistic regression model with a linear term. In addition to the GOSE outcome, we simulated the WLST status of each patient under six scenarios. In Scenario 1, we considered the probability of WLST to be unrelated to the patient characteristics, and the probability of WLST was equal to 0.25 for all patients. In Scenario 2, we assumed that only patients who had a poor 6-month GOSE score could have WLST. In the group with a GOSE score of ≤3, the probability of WLST was 0.35. In Scenario 3, the probability of having WLST was a function of age only. We used a logistic regression model, and the coefficient for age was equal to 1. In Scenario 4, the probability of having WLST was a function of six predictors from Table 1. As with scenario 3, we used a logistic regression model, and the coefficient for each predictor in the logistic regression model was equal to 0.5 (positive or negative depending on the covariate). In Scenario 5, the probability of having WLST was only a function of the extra simulated “unmeasured” variable that was not among the six predictors above. Finally, in Scenario 6, the probability of having WLST was a function of the six predictors from Table 1 and the extra simulated “unmeasured” variable. The simulation code has been included as Supplementary Data.

For each of the 10,000 simulations and WLST scenarios above, we estimated the probability of a good outcome (GOSE score ≥4) using three different statistical methods to account for WLST. For the first statistical method, “complete case,” patients who had WLST were removed from the analysis, and the proportion was estimated based only on the patients with complete data. This is the “complete case” analysis. For the second statistical method, “worst case,” all patients who had WLST were considered to have a poor outcome at 6 months; this is the worst-case imputation analysis. For the third statistical method, “IPW,” we first fit a logistic regression model with WLST as the outcome and the six variables from Table 1 as predictors. Based on this model, we calculated the predicted probability of having WLST for the patients who had WLST and the predicted probability of not having WLST for the patients who did not have WLST. Then, the patients who did not have WLST were weighted based on the inverse of the probability that they did not have WLST. Using these weights, we calculated the probability of a good 6-month outcome using the *svymean* command from the survey library in R.^16^ Since the data were simulated, we also knew the true probability of a good outcome so that we could estimate the bias for the three different statistical methods for handling WLST as the difference between the estimated probability and the true probability. All analyses were completed in the statistical package R version 4.3.0 (www.r-project.org).

## Results

### Simulation results

In Scenario 1 (i.e., WLST occurs at random and is therefore unrelated to patient characteristics), both removing patients from the analysis (complete case) and IPW provided an unbiased estimate of the probability of a good outcome (Table 2). Conversely, the worst-case imputation led to a large underestimate of the probability of a good outcome because some of the patients who experienced WLST would have had a good outcome.

**Table 2.** Estimated Logistic Regression Coefficients for Good Outcome at 6 Months

Simulation scenario	True probability of good outcome (GOSE≥4) in simulated cohort)	Statistical approach for handling WLST	Estimated probability of good outcome (GOSE ≥4) in simulated cohort	Bias (estimated—true)
Scenario 1: WLST occurs at random (i.e., missing outcomes due to WLST are missing completely at random)	0.275	Complete case	0.275	0.000
		Worst case imputation	0.207	−0.068
		IPW	0.276	0.001
Scenario 2: WLST occurs only in patients who would have had a poor 6-month outcome	0.275	Complete case	0.369	0.094
		Worst case imputation	0.275	0.000
		IPW	0.348	0.073
Scenario 3: WLST is only a function of age	0.275	Complete case	0.300	0.025
		Worst case imputation	0.224	−0.051
		IPW	0.275	0.000
Scenario 4: WLST is a function of six measured covariates	0.275	Complete case	0.330	0.055
		Worst case imputation	0.248	−0.027
		IPW	0.276	0.001
Scenario 5: WLST is a function of an unmeasured covariate	0.275	Complete case	0.300	0.025
		Worst case imputation	0.224	−0.051
		IPW	0.288	0.013
Scenario 6: WLST is a function of measured and unmeasured covariates	0.275	Complete case	0.341	0.066
		Worst case imputation	0.254	−0.021
		IPW	0.282	0.007

A positive number for the bias indicates that the approach overestimates the probability of a good outcome, and a negative number for the bias indicates that the approach underestimates the probability of a good outcome.

WLST, withdrawal of life sustaining therapy; GOSE, Glasgow Outcome Scale Extended; IPW, inverse probability weighting.

In Scenario 2 (i.e., only patients who would have had a poor outcome could have WLST), both the complete case analysis and the IPW analysis were biased because these approaches did not require all patients who died after WLST to have a poor outcome (Table 2). Each of these approaches overestimated the probability of a good outcome because each assumed that some patients with WLST would have GOSE ≥4. As expected, the worst-case scenario imputation led to an unbiased estimate of the probability of a good outcome.

In Scenario 3 (i.e., WLST is a function of patient age only) and Scenario 4 (i.e., WLST is a function of six observed covariates from Table 1), the IPW analysis provided the best estimate of the true probability of a good outcome. The complete case analysis overestimated the probability of a good outcome because the patients with WLST were less likely to have a good outcome than those without WLST. Conversely, the worst-case imputation analysis underestimated the probability of a good outcome because some of the patients with WLST had a good outcome. The IPW analysis provided an unbiased estimate when the correct covariates were included in the model for WLST (scenario 4), demonstrating the value of the approach. Further, when the IPW model included six covariates even though only age was associated with WLST (scenario 3), the approach was also unbiased.

In Scenario 5, when the probability of WLST was a function of an unmeasured variable, all three approaches were biased. The complete case analysis and the IPW approach overestimated the probability of a good outcome, and the worst-case imputation underestimated the probability of a good outcome. In Scenario 6, when the probability of WLST was a function of both the measured covariates from Table 1 and the unmeasured covariate, all three approaches were biased, but the IPW approach showed the least bias compared to the other two approaches.

## Discussion

Our simulation study compared three analytic approaches for handling missing outcome data due to WLST after TBI. The results demonstrate that the best approach for estimating outcomes requires understanding how WLST is related to the 6-month outcome or, in statistical terms, the missing data mechanism. To account for WLST, research is needed to determine the predictors of both WLST and the 6-month GOSE outcome so that the missing data mechanism can be characterized and incorporated in the analysis. Properly handling WLST can impact the results of both observational studies and trials.^17^

The three missing data mechanisms defined by Little and Rubin are MCAR, MAR, and not missing at random (NMAR).^5^ MCAR means that the probability of missing data is independent of both the observed and missing data. MAR means that the probability of missing data depends on the observed data but is independent of missing data. NMAR means that the probability of missing data depends on missing data. When the data were MCAR, as in Scenario 1 of our simulation study, both the complete case analysis and the IPW analysis provided an unbiased estimate of the probability of a good outcome. The complete case analysis is valid because the patients who are observed are a random sample of the original group, which means that the analysis of the complete data will be unbiased. Although this result showed that a complete case analysis may be valid in this simulation scenario, it seems unlikely in practice that the patients who die from WLST are a random sample of the original group because these patients likely have a worse prognosis. Our previous work has demonstrated that several features, including age and type of injury, are predictive of WLST, and these features are also likely associated with outcome.^4^ Therefore, the missing data mechanism in TBI studies is unlikely to be MCAR, and a complete case analysis is likely inappropriate.

In Scenario 2, only patients with a poor outcome at 6 months had WLST. In this case, the worst-case imputation approach provides an unbiased estimate of the probability of a good outcome at 6 months because the worst-case imputation is correct. The complete case analysis is biased because the patients with complete data are not a random sample of the original group. The IPW approach is also biased because the patients with WLST are different than patients without WLST in terms of the characteristics included in the IPW model. Therefore, reweighting the observed 6-month outcomes in the patients without WLST using this model to account for those with WLST will not properly account for WLST. However, the worst-case scenario is also clinically unlikely given prior studies suggesting recovery from severe TBI is possible and that at least some patients who died after WLST may have recovered.^2,4,7^

When the data are MAR and a function of patient covariates (Scenarios 3 and 4), IPW can be used to obtain an unbiased estimate of the probability of a good outcome. Further, both the complete case analysis and the worst-case imputation result in biased estimates of the probability of a good outcome. In practice, we anticipate that patient characteristics will predict both WLST and 6-month outcomes. Since the MAR assumption seems likely to be appropriate, the IPW approach will provide an appropriate analysis approach. Importantly, Scenario 3 demonstrates that when the analysis includes several covariates that are not related to WLST in the IPW model, the IPW method still provides an unbiased estimate of the probability of a good outcome.

Scenarios 5 and 6 indicate an important limitation of all three proposed statistical approaches because the missingness was associated with an unobserved characteristic, and this indicates the missingness is NMAR. When the data are NMAR, the patients with WLST will be different than those without WLST, and we cannot account for the missingness using the available information. The results of Scenario 6 showed that the IPW method leads to the least biased estimate when the probability of WLST is a function of both observed and unobserved covariates, demonstrating that this approach may be beneficial even when some of the covariates are unobserved. Scenario 6 represents our current understanding of WLST—there are some demographic and clinical covariates that differentiate patients with and without WLST, but there is also an unmeasured source of variance in who will have WLST. The source of this variance includes unmeasured clinical factors, practitioner or institutional preferences, and cultural and personal beliefs.^18–20^

The results of our study demonstrate the importance of accounting for the missing data mechanism for the estimation of the probability of a good outcome, but our results apply to all clinical studies of TBI. In particular, all clinical studies need to specify the assumptions being made in the statistical analysis, and any potential biases in the estimates from incorrect missing data assumptions should be considered. By clarifying the assumptions of different approaches, the results of clinical studies will be able to be put into proper context.

This study has several limitations that warrant further discussion. First, although the analysis approaches showed different performance across the simulations, our study cannot provide the definitive answer regarding how to handle WLST in statistical analyses because the predictors of WLST and 6-month outcome are still uncertain. Future work will need to continue to refine our understanding to appropriately model WLST. Second, the impact of WLST on estimated treatment effects from clinical trials was not assessed in this study. WLST may impact estimated treatment effects less than prevalence estimates shown in this study if the impact of WLST is the same in both arms of the trial, but clinical trials should consider the missing data mechanism to ensure that assumptions of estimates are understood. Third, our analysis did not assess the impact of other reasons for missingness that might occur in clinical studies. Although this additional type of missingness would require a similar assessment to determine the reasons for missing data, future work is needed for this. A final limitation of this study is that we have estimated the magnitude of the bias based on simulated datasets. The goal of the analysis was to investigate which missing data scenarios and analysis approaches lead to unbiased estimates of the true proportions. It is important to note that the magnitude of the bias reported is a function of the parameters chosen for the simulation. Since these parameters were not known, the magnitude of the bias should not be the focus, but our approach indicates when each analysis approach is unbiased. Two additional simulations were completed with a higher and lower proportion of subjects having WLST, and the results were qualitatively similar to those presented in this article, but the estimated bias was different.

## Conclusions

In conclusion, if we are willing to assume that some of the patients who die of WLST might have had a good outcome at 6 months, the appropriate approach for handling WLST in TBI studies requires careful consideration of the missing data mechanism. If the missing data mechanism is known, the appropriate analysis approach can be chosen. If the missing data mechanism is unknown, investigators should ensure that they collect as many patient characteristics as possible so that the MAR assumption is likely to hold.

## Abbreviations Used

GCS: Glasgow Coma Scale
GOSE: Glasgow Outcome Scale-Extended
IPW: Inverse probability weighting
LST: life sustaining therapy
MCAR: Missing completely at random
MAR: Missing at random
NMAR: Not missing at random
TBI: Traumatic brain injury
WLST: withdrawal of life sustaining therapy
