# Supplementary material for: Accounting for Withdrawal of Life-Sustaining Treatment in the Analysis of Traumatic Brain Injury Studies
Source: Neurotrauma Rep. 2025 May 27;6(1):435–41. doi: 10.1089/neur.2025.0010 (PMC12171701; doi:10.1089/neur.2025.0010)
Supplement: Supplementary Data [file neur.2025.0010_supplementary_data.docx]

##Simulation for the paper

set.seed(12345)

numsim<-10000

results<-matrix(NA,10000,24)

names(results)<-c("true_outcome1","observed_outcome1","bad_outcome1","obs_weighted_glm1",

"true_outcome2","observed_outcome2","bad_outcome2","obs_weighted_glm2",

"true_outcome3","observed_outcome3","bad_outcome3","obs_weighted_glm3",

"true_outcome4","observed_outcome4","bad_outcome4","obs_weighted_glm4",

"true_outcome5","observed_outcome5","bad_outcome5","obs_weighted_glm5",

"true_outcome6","observed_outcome6","bad_outcome6","obs_weighted_glm6")

numsubj<-500

library(weights)

library(survey)

for (i in 1:numsim){

wlst<-data.frame(ID=c(1:numsubj))

wlst$group<-ifelse(wlst$ID<=numsubj/4,1,0)

wlst$age<-ifelse(wlst$group==1,rnorm(numsubj,mean=59.2, sd=17.9),rnorm(numsubj,mean=42.5, sd=18.1))

wlst$agez<-(wlst$age-mean(wlst$age))/sd(wlst$age)

wlst$day0gcs<-ifelse(wlst$group==1,rnorm(numsubj,mean=2.89, sd=1.60),rnorm(numsubj,mean=5.36, sd=1.26))

wlst$day0gcsz<-(wlst$day0gcs-mean(wlst$day0gcs))/sd(wlst$day0gcs)

wlst$depprob<-ifelse(wlst$group==1,0.44, 0.14)

wlst$dep<-ifelse(wlst$depprob>runif(numsubj,0,1),1,0)

wlst$comp<-ifelse(wlst$group==1,rnorm(numsubj,mean=3.38, sd=3.15),rnorm(numsubj,mean=1.45, sd=2.41))

wlst$compz<-(wlst$comp-mean(wlst$comp))/sd(wlst$comp)

wlst$iss<-ifelse(wlst$group==1,rnorm(numsubj,mean=26.9, sd=11.3),rnorm(numsubj,mean=19.4, sd=11.0))

wlst$issz<-(wlst$iss-mean(wlst$iss))/sd(wlst$iss)

wlst$ctr<-ifelse(wlst$group==1,rnorm(numsubj,mean=4.22, sd=1.39),rnorm(numsubj,mean=2.79, sd=1.02))

wlst$ctrz<-(wlst$ctr-mean(wlst$ctr))/sd(wlst$ctr)

wlst$other<-ifelse(wlst$group==1,rnorm(numsubj,mean=59.2, sd=17.9),rnorm(numsubj,mean=42.5, sd=18.1))

wlst$otherz<-(wlst$other-mean(wlst$other))/sd(wlst$other)

wlst$outcomeprob<-exp((log(1/4))-0.5*wlst$agez+0.5*wlst$day0gcsz-0.5*wlst$dep-0.5*wlst$compz-0.5*wlst$issz-0.5*wlst$ctrz-0.5*wlst$otherz)/

(1+exp((log(1/4))-0.5*wlst$agez+0.5*wlst$day0gcsz-0.5*wlst$dep-0.5*wlst$compz-0.5*wlst$issz-0.5*wlst$ctrz-0.5*wlst$otherz))

wlst$outcome<-ifelse(wlst$outcomeprob>runif(numsubj,0,1),1,0)

##Scenario 1

wlst$obs_wlst1<-ifelse(0.25>runif(numsubj,0,1),1,0)

wlst$obs_outcome1<-ifelse(wlst$obs_wlst1==1,NA,wlst$outcome)

wlst$badimp_outcome1<-ifelse(wlst$obs_wlst1==1,0,wlst$outcome)

logreg_mod1<-glm(obs_wlst1~agez+day0gcsz+dep+compz+issz+ctrz,data=wlst,family=binomial)

wlst$prob_wlst1<-predict(logreg_mod1,data=wlst,type="response")

wlst$weight_wlst1<-ifelse(wlst$obs_wlst1==1,NA,1/(1-wlst$prob_wlst1))

results[i,1]<-prop.table(table(wlst$outcome))[2]

results[i,2]<-prop.table(table(wlst$obs_outcome1))[2]

results[i,3]<-prop.table(table(wlst$badimp_outcome1))[2]

wlst1 <- svydesign(id=~ID, weights=~weight_wlst1, data=wlst[!is.na(wlst$weight_wlst1),])

results[i,4]<-svymean(~obs_outcome1, wlst1)[1]

##Scenario 2

wlst$obs_wlst2<-0

wlst$obs_wlst2[wlst$outcome==0]<-ifelse(0.35>runif(length(wlst$obs_wlst2[wlst$outcome==0]),0,1),1,0)

wlst$obs_outcome2<-ifelse(wlst$obs_wlst2==1,NA,wlst$outcome)

wlst$badimp_outcome2<-ifelse(wlst$obs_wlst2==1,0,wlst$outcome)

logreg_mod2<-glm(obs_wlst2~agez+day0gcsz+dep+compz+issz+ctrz,data=wlst,family=binomial)

wlst$prob_wlst2<-predict(logreg_mod2,data=wlst,type="response")

wlst$weight_wlst2<-ifelse(wlst$obs_wlst2==1,NA,1/(1-wlst$prob_wlst2))

results[i,5]<-prop.table(table(wlst$outcome))[2]

results[i,6]<-prop.table(table(wlst$obs_outcome2))[2]

results[i,7]<-prop.table(table(wlst$badimp_outcome2))[2]

wlst2 <- svydesign(id=~ID, weights=~weight_wlst2, data=wlst[!is.na(wlst$weight_wlst2),])

results[i,8]<-svymean(~obs_outcome2, wlst2)[1]

##Scenario 3

wlst$wlst_prob3<-exp(-1.3+1*wlst$agez)/(1+exp(-1.3+1*wlst$agez))

wlst$obs_wlst3<-ifelse(wlst$wlst_prob3>runif(numsubj,0,1),1,0)

wlst$obs_outcome3<-ifelse(wlst$obs_wlst3==1,NA,wlst$outcome)

wlst$badimp_outcome3<-ifelse(wlst$obs_wlst3==1,0,wlst$outcome)

logreg_mod3<-glm(obs_wlst3~agez+day0gcsz+dep+compz+issz+ctrz,data=wlst,family=binomial)

wlst$prob_wlst3<-predict(logreg_mod3,data=wlst,type="response")

wlst$weight_wlst3<-ifelse(wlst$obs_wlst3==1,NA,1/(1-wlst$prob_wlst3))

results[i,9]<-prop.table(table(wlst$outcome))[2]

results[i,10]<-prop.table(table(wlst$obs_outcome3))[2]

results[i,11]<-prop.table(table(wlst$badimp_outcome3))[2]

wlst3 <- svydesign(id=~ID, weights=~weight_wlst3, data=wlst[!is.na(wlst$weight_wlst3),])

results[i,12]<-svymean(~obs_outcome3, wlst3)[1]

##Scenario 4

wlst$wlst_prob4<-exp(-1.6+0.5*wlst$agez-0.5*wlst$day0gcsz+ 0.5*wlst$dep+0.5*wlst$compz+ 0.5*wlst$issz+0.5*wlst$ctrz)/(1+exp(-1.6+0.5*wlst$agez-0.5*wlst$day0gcsz+ 0.5*wlst$dep+0.5*wlst$compz+ 0.5*wlst$issz+0.5*wlst$ctrz))

wlst$obs_wlst4<-ifelse(wlst$wlst_prob4>runif(numsubj,0,1),1,0)

wlst$obs_outcome4<-ifelse(wlst$obs_wlst4==1,NA,wlst$outcome)

wlst$badimp_outcome4<-ifelse(wlst$obs_wlst4==1,0,wlst$outcome)

logreg_mod4<-glm(obs_wlst4~agez+day0gcsz+dep+compz+issz+ctrz,data=wlst,family=binomial)

wlst$prob_wlst4<-predict(logreg_mod4,data=wlst,type="response")

wlst$weight_wlst4<-ifelse(wlst$obs_wlst4==1,NA,1/(1-wlst$prob_wlst4))

results[i,13]<-prop.table(table(wlst$outcome))[2]

results[i,14]<-prop.table(table(wlst$obs_outcome4))[2]

results[i,15]<-prop.table(table(wlst$badimp_outcome4))[2]

wlst4 <- svydesign(id=~ID, weights=~weight_wlst4, data=wlst[!is.na(wlst$weight_wlst4),])

results[i,16]<-svymean(~obs_outcome4, wlst4)[1]

##Scenario 5

wlst$wlst_prob5<-exp(-1.3+1*wlst$otherz)/(1+exp(-1.3+1*wlst$otherz))

wlst$obs_wlst5<-ifelse(wlst$wlst_prob5>runif(numsubj,0,1),1,0)

wlst$obs_outcome5<-ifelse(wlst$obs_wlst5==1,NA,wlst$outcome)

wlst$badimp_outcome5<-ifelse(wlst$obs_wlst5==1,0,wlst$outcome)

logreg_mod5<-glm(obs_wlst5~agez+day0gcsz+dep+compz+issz+ctrz,data=wlst,family=binomial)

wlst$prob_wlst5<-predict(logreg_mod5,data=wlst,type="response")

wlst$weight_wlst5<-ifelse(wlst$obs_wlst5==1,NA,1/(1-wlst$prob_wlst5))

results[i,17]<-prop.table(table(wlst$outcome))[2]

results[i,18]<-prop.table(table(wlst$obs_outcome5))[2]

results[i,19]<-prop.table(table(wlst$badimp_outcome5))[2]

wlst5 <- svydesign(id=~ID, weights=~weight_wlst5, data=wlst[!is.na(wlst$weight_wlst5),])

results[i,20]<-svymean(~obs_outcome5, wlst5)[1]

##Scenario 6

wlst$wlst_prob6<-exp(-1.8+0.5*wlst$agez-0.5*wlst$day0gcsz+0.5*wlst$dep+0.5*wlst$compz+0.5*wlst$issz+0.5*wlst$ctrz+1*wlst$otherz)/

(1+exp(-1.8+0.5*wlst$agez-0.5*wlst$day0gcsz+0.5*wlst$dep+0.5*wlst$compz+0.5*wlst$issz+0.5*wlst$ctrz+1*wlst$otherz))

wlst$obs_wlst6<-ifelse(wlst$wlst_prob6>runif(numsubj,0,1),1,0)

wlst$obs_outcome6<-ifelse(wlst$obs_wlst6==1,NA,wlst$outcome)

wlst$badimp_outcome6<-ifelse(wlst$obs_wlst6==1,0,wlst$outcome)

logreg_mod6<-glm(obs_wlst6~agez+day0gcsz+dep+compz+issz+ctrz,data=wlst,family=binomial)

wlst$prob_wlst6<-predict(logreg_mod6,data=wlst,type="response")

wlst$weight_wlst6<-ifelse(wlst$obs_wlst6==1,NA,1/(1-wlst$prob_wlst6))

results[i,21]<-prop.table(table(wlst$outcome))[2]

results[i,22]<-prop.table(table(wlst$obs_outcome6))[2]

results[i,23]<-prop.table(table(wlst$badimp_outcome6))[2]

wlst6 <- svydesign(id=~ID, weights=~weight_wlst6, data=wlst[!is.na(wlst$weight_wlst6),])

results[i,24]<-svymean(~obs_outcome6, wlst6)[1]

}

apply(results,2,mean)
